# Comparison of AMG 416 and cinacalcet in rodent models of uremia

**DOI:** 10.1186/1471-2369-15-81

**Published:** 2014-05-19

**Authors:** Sarah Walter, Amos Baruch, Shawn T Alexander, Julie Janes, Eiketsu Sho, Jin Dong, Qun Yin, Derek Maclean, Dirk B Mendel, Felix Karim, Randolph M Johnson

**Affiliations:** 1 Amgen Inc, 1120 Veterans Blvd., South San Francisco, CA 94080, USA; 2 Present address: Labrys Biologics, San Mateo, CA, USA; 3 Present address: Genentech, South San Francisco, CA, USA; 4 Present address: Calithera Biosciences, South San Francisco, CA, USA; 5 Present address: Kunming Biomedical, Kunming, China; 6 Present address: MedImmune, Hayward, CA, USA; 7 Present address: Sutro Biopharma, South San Francisco, CA, USA; 8 Present address: MedImmune, Gaithersburg, MD, USA

**Keywords:** Calcium-sensing receptor, Secondary hyperparathyroidism (SHPT), Chronic kidney disease (CKD), Uremic rat model, AMG 416

## Abstract

**Background:**

AMG 416 is a novel peptide agonist of the calcium-sensing receptor (CaSR). This report describes the activity of AMG 416 in two different rodent models of uremia, compared in each case to cinacalcet, an approved therapeutic for secondary hyperparathyroidism (SHPT) in patients with chronic kidney disease on dialysis.

**Methods:**

AMG 416 was administered as a single intravenous (IV) bolus in a severe, acute model of renal insufficiency (the “1K1C” model) and plasma parathyroid hormone (PTH) and serum calcium levels were monitored for 24 hours. In a chronic, less severe model of renal dysfunction, the 5/6 nephrectomy (5/6 Nx) model, AMG 416 was administered as a once-daily IV bolus for 28 days. Both studies included a control (vehicle) group and a comparison cinacalcet group (po dosing at 30 mg/kg and 10 mg/kg for the 1K1C and 5/6 Nx studies, respectively).

**Results:**

Administration of AMG 416 by IV bolus injection into rats with acute renal dysfunction (1K1C model) resulted in a sustained reduction in plasma PTH from the initial elevated values. Following a single IV bolus (0.5 mg/kg), AMG 416 caused a substantial drop in PTH levels which remained approximately 50% below their initial level at 24 hrs. In the same model, oral treatment with cinacalcet (30 mg/kg) resulted in an acute drop in PTH which almost returned to the starting level by 24 hours after dosing. In the 5/6 Nx chronic uremia model, daily IV dosing of AMG 416 over 4 weeks (1 mg/kg) resulted in a sustained reduction in PTH, with approximately 50% of the initial level observed 48 hours post treatment throughout the study. Cinacalcet treatment (10 mg/kg) in the same model resulted in acutely lowered plasma PTH levels which returned to placebo levels by 24 hours post-dose. Consistent with the reductions in plasma PTH, reductions in serum calcium were observed in both AMG 416- and cinacalcet-treated animals.

**Conclusions:**

As a long-acting CaSR agonist suitable for administration by the IV route, AMG 416 is a potential new therapy for the treatment of CKD patients with SHPT receiving hemodialysis.

## Background

Chronic kidney disease (CKD) is a condition characterized by a gradual loss of kidney function. As a consequence of reduced renal function, normal mineral regulatory mechanisms are disrupted. CKD is often further complicated by the development of secondary hyperparathyroidism (SHPT) due to these disturbances in mineral metabolism [1]. Increased PTH secretion in response to hypocalcemia is mediated by the calcium-sensing receptor (CaSR) a G-protein coupled receptor (GPCR) located on the parathyroid glands [2].

The use of the calcimimetic agent cinacalcet (Sensipar®/Mimpara®) [3] has represented an advance in the management of patients with SHPT receiving dialysis. Cinacalcet is an allosteric modulator of the CaSR that sensitizes the receptor to extracellular calcium, resulting in reduced PTH secretion from the parathyroid gland [4]. The decrease in PTH is accompanied by reductions in serum calcium and phosphorus levels in patients with SHPT receiving dialysis [3].

AMG 416 is a novel peptide agonist of the CaSR that is being developed as an intravenous (IV) product for the treatment of CKD with SHPT. In a recent publication, we showed that AMG 416 is effective at reducing plasma PTH in preclinical uremic rat studies, modifying parathyroid gland receptor levels and impacting calcium and phosphorus levels [5]. AMG 416 has also proven effective in clinical studies in both normal healthy males and CKD patients with SHPT receiving hemodialysis [6,7]. With the IV route of administration, AMG 416 is anticipated to have improved compliance relative to cinacalcet, and offers the potential for improved tolerability. In this paper, we sought to directly compare the efficacy of these two compounds in two different uremic rodent models. We chose a quite severe, acute model of renal insufficiency (the 1 kidney removal and 1 clip, or “1K1C” model) to examine the efficacy of a single IV dose of AMG 416 vs oral cinacalcet in the presence of elevated PTH and serum creatinine. In addition, we examined the activity of AMG 416 in a model of chronic uremia, in rats under 5/6 nephrectomy in order to compare the efficacy of AMG 416 and cinacalcet over repeated dosing during a 28-day administration period.

## Methods

### Test compounds

AMG 416 was prepared as described previously [5]. Cinacalcet was prepared from commercial tablets as a suspension in normal saline.

### Evaluation of AMG 416 in “1 Kidney 1 Clip” (1K1C) rat model of acute renal dysfunction

The 1K1C model was based on the original model developed by Goldblatt [8]. Male Sprague Dawley (SD) rats (~300 g) were purchased from Charles River Laboratories (CRL), pre-cannulated in the jugular and femoral veins for blood sampling and dosing, respectively. The study protocol was approved by the Institutional Animal Care and Use Committee (IACUC) of KAI Pharmaceuticals, Inc. General anesthesia was induced and maintained by intraperitoneal (IP) injection of sodium pentobarbital (5.2%, 0.4 mL/rat). Both kidneys were exposed via laparotomy. The right kidney was removed following ligation of the right renal pedicle and ureter. A microvascular clip was applied to the left renal artery for 45 min and then removed. Ischemia was assessed by color change in the affected kidney. The abdominal incision was then closed and the animal was allowed to recover for approximately 48 hrs prior to dosing. Animals were treated with either AMG 416 (0.5 mg/kg in 0.5 mL, IV; n = 5), saline (0.5 mL, IV; n = 3) or cinacalcet (30 mg/kg, 1 mL po; n = 6). Animals were given free access to food and water. Blood samples (~0.2 mL) were taken from the jugular cannula under anesthesia (2% isoflurane in O_2_). Samples were taken at the indicated times and processed immediately for plasma.

### Evaluation of AMG 416 in 5/6 Nephrectomized Rats

A 4-week, repeat dose study was performed in 5/6 nephrectomized male SD rats at CRL (Wilmington, MA). The protocol was approved by the IACUC of CRL. Animals weighed ~300 g at the time of the first surgery. In the first operation, 2/3 of one kidney was surgically removed. After a one-week recovery, the other kidney was removed, leaving the rat with 1/6 of its original renal capacity. Catheters were implanted in each jugular vein during the second operation for drug administration and blood sampling. Animals were allowed to recover for 9-10 days following surgery prior to dosing. Thirty-six animals were included in the study. Animals were randomized to study drug based on serum creatinine and plasma PTH collected on Days -3 and -2 (~7 days post final surgery; data averaged to determine “pre-dose” value). Twelve animals per group received daily administration of saline (0.5 mL, IV bolus) or AMG 416 (1 mg/kg in 0.5 mL, IV bolus) or cinacalcet (10 mg/kg in 1 mL, oral gavage) for 28 days. Blood samples (0.45 mL; tail vein) were taken for PTH and calcium analysis prior to dosing and at 6 and 16 hrs post-dose on Days 7, 14, 21 and 28. Animals were sacrificed on Day 30 (48 hrs after the last dose) and blood was taken for PTH analysis. Due to mortality during the study, group sizes for PTH and Ca analysis were 7, 6 and 9 for saline, cinacalcet and AMG 416, respectively.

### Plasma and serum analysis

Plasma PTH levels were quantified according to the manufacturer’s protocol using rat (1-84) bioactive intact PTH ELISA kits from Immutopics International (Rat: #60-2700; San Clemente, California). Raw data were analyzed with GraphPad Prism (GraphPad, La Jolla, California). When appropriate, one-way ANOVA was used to determine statistical significance with Bonferroni post-test analysis.

Serum was obtained by allowing blood samples (0.2 – 0.3 mL) to clot for approximately 30-60 minutes followed by centrifugation. Creatinine concentration was determined according to the manufacturer’s protocol using the QuantiChrom™ kit, (DICT-500; BioAssay Systems, Hayward, CA). Serum samples were analyzed for total calcium content at SRI (Menlo Park, CA) using the Roche Cobas C-501 autoanalyzer.

## Results

### AMG 416 suppresses PTH in animals with severely compromised renal function

**Figure 1:**
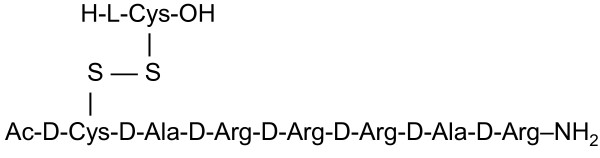
Structure of AMG 416.

**Figure 2:**
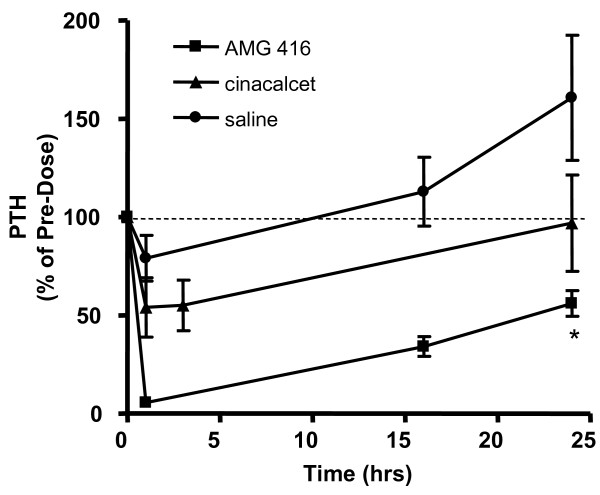
**AMG 416 administration lowers PTH in a severe model of kidney dysfunction.** Rats subjected to IKIC surgery were treated with a single dose of either: AMG 416 by IV bolus injection (0.5 mg/kg), cinacalcet by po administration (30 mg/kg), or saline IV bolus (0.5 mL). Serum creatinine levels (mg/dL) for the three groups were: AMG 416 (5.5), cinacalcet (5.1), saline (6.1). Blood samples were removed at indicated times to measure plasma PTH. Mean group baseline PTH levels (pg/mL) prior to dosing were: AMG 416 (667), cinacalcet (949), saline (1026). Data are presented as group mean ± SEM (AMG 416, n = 5; cinacalcet n = 6; saline, n = 3). *, p < 0.05 vs. saline.

To mimic the pathology of renal failure and the associated increases in PTH that are seen in ESRD patients with chronic kidney disease, mineral and bone disorder (CKD-MBD), AMG 416 (Figure 1) was evaluated in a rat model of acute renal insufficiency (“1K1C” model). Unlike the less severe 5/6 nephrectomy model, animals subjected to this procedure have almost no kidney function, and highly elevated serum creatinine and plasma PTH levels (group mean of 5.4 mg/dL and 865 pg/mL respectively); in these ways the model parallels ESRD. Forty-eight hours after surgery, animals were administered a single IV bolus of AMG 416 (0.5 mg/kg), saline or a single po dose of cinacalcet (30 mg/kg). Administration of AMG 416 resulted in a rapid and substantial (>90%) reduction in plasma PTH within 1-2 hr (Figure 2). This reduction in plasma PTH levels was maintained over the entire 24 hr monitoring period (p < 0.05 vs saline at 24 hr). Treatment with cinacalcet also lowered plasma PTH in this model, consistent with previous reports [8], but to a lesser extent and for a shorter duration than seen with AMG 416. Animals treated with vehicle showed no reduction in PTH during the study.

### AMG 416 treatment results in prolonged PTH suppression in a rat model of chronic kidney disease

AMG 416 was also evaluated in a rat model of chronic kidney disease (the 5/6 nephrectomy model or 5/6 Nx), a commonly used model of CKD-MBD that enables chronic dosing [9]. Approximately 1 week after completion of the 5/6 Nx surgery, animals were randomized based upon PTH and serum creatinine values to one of three treatment groups: saline (IV), cinacalcet (10 mg/kg po) or AMG 416 (1 mg/kg IV). Animals were dosed daily for 28 days.

**Figure 3:**
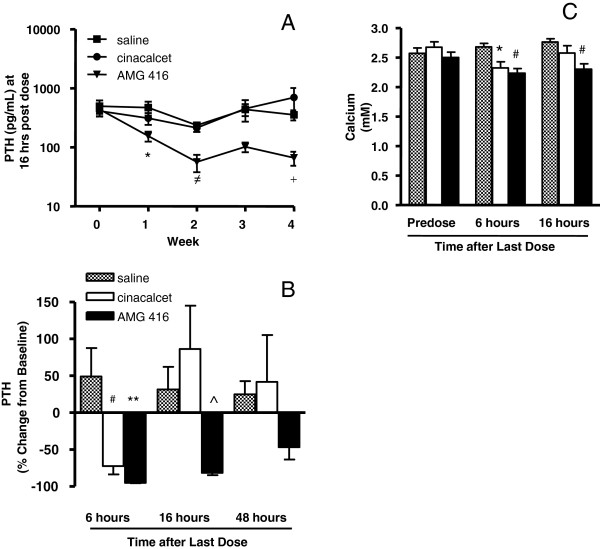
**Repeat administration of AMG 416 results in extended reduction of PTH in renally compromised rats.** Rats subjected to 5/6 nephrectomy were treated daily for 28 days with either: AMG 416 (1 mg/kg administered by IV bolus), saline (IV bolus) or cinacalcet (10 mg/kg administered po). Blood chemistries are reported relative to the last (day 28) dose. **(A)** Plasma PTH measured at 6, 16 and 48 hr from the last dose. **(B)** Plasma PTH measured at 16 hr following dosing on Days 7, 14, 21 and 28. **(C)** Serum calcium at pre-dose, 6 and 16 h from the last dose. Data are presented as group mean ± SEM (Saline [hatched bars] n = 7; cinacalcet [white bars] n = 6; AMG 416 [black bars] n = 9). *, p < 0.05 vs. saline; #, p < 0.01 vs. saline; **, p < 0.001 vs. saline; ^, p < 0.01 vs. cinacalcet; ≠, p < 0.001 vs. cinacalcet, saline; +, p < 0.05 vs. cinacalcet.

Prior to dosing, mean plasma PTH levels in all three dose groups were similar (400 - 500 pg/mL), and elevated in comparison to normal animals (~150 pg/mL). Serum creatinine levels were also increased with group mean values of 1.4, 1.3 and 1.3 mg/dL for the saline, cinacalcet and AMG 416 groups, respectively. During the study, PTH levels were measured pre-dose and 6 and 16 hr after dosing on Days 7, 14, 21 and 28, and also 48 hr after the final dose (Day 28). In the saline-treated group, plasma PTH levels fluctuated over the 4-week study but generally remained at or above the 400-500 pg/mL starting level (Figure 3A). In contrast, acute reductions in plasma PTH levels were observed in both cinacalcet- and AMG 416-treated animals (Table 1). This is shown in Table 1 and Figure 3B, where six hours after the last dose on Day 28, plasma PTH was reduced by approximately 70-90% from baseline in both the AMG 416- and cinacalcet-treated groups; however, by 16 hr post dosing, plasma PTH had returned to pre-dose levels in the cinacalcet animals, comparable to levels seen in saline-treated animals. In contrast, for AMG 416-treated animals, PTH levels remained suppressed at the 16 hr time point and were still 40-50% reduced from baseline values at 48 hr following dosing (on Day 28). As shown in Figure 3A, PTH was consistently lower for AMG 416-treated rats at 16 hr post dosing than for the cinacalcet or vehicle groups throughout the 4 weeks of treatment.

**Table 1:** PTH (pg/mL) and Ca (mg/dL) levels in 5/6 Nx repeat-dose study

**PTH**				
**Week**	**Hour post dosing**	**Cinacalcet**	**AMG 416**	**Vehicle**
Week 1	6 hr	128 ± 44	30 ± 6	758 ± 191
	16 hr	312 ± 72	155 ± 30	472 ± 126
Week 4	6 hr	115 ± 73	18 ± 4	451 ± 162
	16 hr	700 ± 315	66 ± 18	357 ± 69
**Calcium**				
**Week**	**Hour post dosing**	**Cinacalcet**	**AMG 416**	**Vehicle**
Week 1	6 hr	8.9 ± 0.3	8.0 ± 0.3	10.4 ± 0.3
	16 hr	10.0 ± 0.3	8.5 ± 0.3	11.0 ± 0.3
Week 4	6 hr	9.3 ± 0.4	8.9 ± 0.3	10.7 ± 0.2
	16 hr	10.3 ± 0.5	9.2 ± 0.4	11.1 ± 0.2

Data shown are group means ± SEM.

Consistent with the reductions in plasma PTH, modest reductions in serum calcium were observed in both AMG 416- and cinacalcet-treated animals at 6 hr post dosing and, consistent with the prolonged reductions in PTH, were still reduced in the AMG 416 animals at 16 hr post dosing (Figure 3C).

## Discussion

The 1K1C model is a severe, acute model of renal dysfunction which enables the activity of AMG 416 and cinacalcet to be investigated in the presence of the highly elevated levels of PTH and lack of kidney function typically seen in CKD-MBD patients receiving hemodialysis [1]. Due to its acute nature, the 1K1C model is not associated with the parathyroid gland hyperplasia seen in the rat 5/6 Nx uremic model and in dialysis patients with SHPT [9,10]. However, it is an excellent model for assessing PTH-lowering activity in the background of severe kidney dysfunction. In this study, plasma PTH levels were significantly reduced by a single dose of either AMG 416 or cinacalcet. The effect of cinacalcet on PTH was of a lesser extent and shorter duration than seen with AMG 416, which maintained PTH lowering for more than 24 hr. The prolonged suppression of plasma PTH in the 1K1C model by AMG 416 is consistent with the pharmacokinetics (PK) observed for AMG 416 in normal rats and in different uremic models [5]. The absence of kidney function results in an extended terminal half life for AMG 416, indicating that the kidney is a major clearance organ for the peptide [5]. In contrast, the half-life of cinacalcet is independent of kidney function [11], as the main route of clearance is through hepatic mechanisms [12]. Consistent with the animal data and modeling PK studies, IV administration of AMG 416 to hemodialysis patients with SHPT resulted in dose-dependent, sustained control of PTH throughout the interdialytic period [6].

There are a number of endpoints that can be examined in preclinical models of SHPT such as effects on PTH, serum phosphorus and calcium levels and parathyroid gland hyperplasia. In these studies, we focused on how AMG 416 compared with an approved calcimimetic, cinacalcet, at lowering plasma PTH. Additional rodent studies document the changes to parathyroid gland biology that can take place with chronic AMG 416 treatment [5], and the molecule’s effects on serum phosphorus levels have been shown in the clinical setting [6,13]. For technical reasons it was not possible to obtain data for serum phosphorus in these studies.

The dose of cinacalcet in the chronic study was chosen based upon PK exposure data in rats since the area under the curve (AUC) was comparable to CKD-MBD patients with SHPT receiving hemodialysis receiving the 60 mg dose [9,14] and is consistent with a number of published studies using cinacalcet [9,15]. The resulting pharmacodynamic behavior for cinacalcet in this study is also consistent with previously published results [9]. Although treatment with both agents reduced PTH shortly after dosing (6 hr post treatment), only AMG 416 was associated with sustained PTH reductions throughout and beyond the dosing interval. Throughout the four weeks of treatment in this study, animals treated with AMG 416 maintained a consistent, lower level of plasma PTH when compared with cinacalcet or placebo groups. These longer-term effects may in part arise from reversal of abnormal parathyroid gland physiology, as observed in other studies with AMG 416 [5].

In addition to attenuating plasma PTH in both models, both cinacalcet and AMG 416 caused a decrease in serum calcium, in agreement with previously reported studies with cinacalcet treatment in normal and uremic rats [4,9] as well as in dialysis patients with SHPT [3], and consistent with the known pharmacological action of lowering PTH with this class of therapies.. This reduction in calcium can persist beyond the PTH lowering effect. In response to reduced serum calcium, a “rebound” in PTH can occur as the body perceives a state of hypocalcemia. This is seen in Table 1 and Figure 3B, where the cinacalcet-treated animals show an increased PTH level over baseline at the 16 hour time point.

## Conclusions

Taken together, these findings demonstrate that AMG 416 suppresses plasma PTH and is a potential new therapy for the treatment of CKD patients with SHPT receiving hemodialysis. Because AMG 416 is administered by the IV route, it is anticipated that it may have improved efficacy and superior compliance compared with cinacalcet in this indication [16]. In addition, AMG 416 offers the potential for improved gastrointestinal tolerability over cinacalcet [3,17], and because it is not metabolized by the liver and does not interact with P450 enzymes [17], avoids the risk of P450-mediated drug-drug interactions.

## Competing interests

S. Alexander, D. Maclean and F. Karim are employees of Amgen and receive salary and/or own or have an interest in Amgen stock. The other authors have no conflicting interests.

## Authors’ contributions

SW wrote the manuscript and contributed to the design of the studies. AB conceived of the studies and aided in data interpretation. SA performed the 1K1C study and PTH analysis. JJ performed the 1K1C study. ES optimized the 1K1C surgical procedure. JD performed the 1K1C surgical procedure. QY synthesized peptides used for the studies. DM contributed to study design and writing of the manuscript. DBM oversaw study design and interpretation of data. FK oversaw study design, data interpretation and contributed to the writing of the manuscript. RJ oversaw study design. All authors read and approved the final manuscript.

## Pre-publication history

The pre-publication history for this paper can be accessed here:

http://www.biomedcentral.com/1471-2369/15/81/prepub
